# Calcium Ions in the Physiology and Pathology of the Central Nervous System

**DOI:** 10.3390/ijms252313133

**Published:** 2024-12-06

**Authors:** Damian Pikor, Mikołaj Hurła, Bartosz Słowikowski, Oliwia Szymanowicz, Joanna Poszwa, Natalia Banaszek, Alicja Drelichowska, Paweł P. Jagodziński, Wojciech Kozubski, Jolanta Dorszewska

**Affiliations:** 1 Laboratory of Neurobiology, Department of Neurology, Poznan University of Medical Sciences, 60-355 Poznan, Poland; pikor.pikor7@gmail.com (D.P.); mikolaj.hurla@gmail.com (M.H.); 76624@student.ump.edu.pl (O.S.); jposzwa@ump.edu.pl (J.P.); nata.banaszek@wp.pl (N.B.); drelichowskaalicja@gmail.com (A.D.); 2 Department of Biochemistry and Molecular Biology, Poznan University of Medical Sciences, 60-781 Poznan, Poland; bslowikowski@ump.edu.pl (B.S.); pjagodzi@ump.edu.pl (P.P.J.); 3 Chair and Department of Neurology, Poznan University of Medical Sciences, 60-355 Poznan, Poland; wkozubski@ump.edu.pl

**Keywords:** calcium ions, calcium channels, channelopathies, neurodegenerative processes, neurological diseases

## Abstract

Calcium ions play a key role in the physiological processes of the central nervous system. The intracellular calcium signal, in nerve cells, is part of the neurotransmission mechanism. They are responsible for stabilizing membrane potential and controlling the excitability of neurons. Calcium ions are a universal second messenger that participates in depolarizing signal transduction and contributes to synaptic activity. These ions take an active part in the mechanisms related to memory and learning. As a result of depolarization of the plasma membrane or stimulation of receptors, there is an extracellular influx of calcium ions into the cytosol or mobilization of these cations inside the cell, which increases the concentration of these ions in neurons. The influx of calcium ions into neurons occurs via plasma membrane receptors and voltage-dependent ion channels. Calcium channels play a key role in the functioning of the nervous system, regulating, among others, neuronal depolarization and neurotransmitter release. Channelopathies are groups of diseases resulting from mutations in genes encoding ion channel subunits, observed including the pathophysiology of neurological diseases such as migraine. A disturbed ability of neurons to maintain an appropriate level of calcium ions is also observed in such neurodegenerative processes as Alzheimer’s disease, Parkinson’s disease, Huntington’s disease, and epilepsy. This review focuses on the involvement of calcium ions in physiological and pathological processes of the central nervous system. We also consider the use of calcium ions as a target for pharmacotherapy in the future.

## 1. Introduction

Calcium ions (Ca^2+^) are the ionized form of calcium with an atomic number of 20. They are formed when a calcium atom loses two electrons, resulting in a positively charged +2 ion. Maintaining calcium ion homeostasis is crucial for sustaining optimal conditions for physiological processes occurring in the nervous system. They can occur in various forms, the most common of which is free Ca^2+^. They are involved in a number of processes in the nervous system such as neurotransmission, synaptic plasticity, regulation of intracellular transmission, apoptosis, and action potentials. On the other hand, disruption of calcium ion homeostasis leading to an excessive influx of Ca^2+^ into cells can lead to apoptosis and excitotoxicity resulting in nerve cell damage. There are specific proteins like parvalbumin and calreticulin, which bind excess Ca^2+^ inside nerve cells and are responsible for maintaining optimal intracellular calcium levels. Ca^2+^ are also crucial for muscle contraction mechanisms, including those controlled by the nervous system. Their influx into the cells of motor neurons results in the release of acetylcholine, which enables smooth muscle contraction of the autonomic nervous system. Ca^2+^ are also found bound to proteins such as calmodulin, which activates a calcium-dependent protein kinase that regulates processes related to memory and learning. Another example of a calcium-binding protein (CaBPs) is synaptotagmin 9 (SYT9), which, along with calcium ions, plays an important role in the release of neurotransmitters into the synaptic gap [1].

Ca^2+^ are involved in the pathological processes underlying many neurological diseases, such as Alzheimer’s disease (AD), Parkinson’s disease (PD), Huntington’s disease (HD), epilepsy, and migraine. In AD, Ca^2+^ promote the formation of β-amyloid (Aβ) plaques (APs) and neurofibrillary tangles (NFTs) by affecting amyloid precursor processing (APP) pathways, leading to increased production of Aβ [2]. Moreover, they contribute to increased oxidative stress and excessive activation of microglia, which also underlie neurodegenerative processes that lead to the development of the disease [3]. In PD, mitochondrial function is impaired, which can result in abnormal regulation of calcium ion concentrations and consequently potentiate oxidative stress and apoptosis. Mitochondria, acting as dynamic calcium buffers, play an important role in maintaining calcium homeostasis by protecting cells from calcium overload by storing Ca^2+^ when their level in the cytoplasm is too high [4]. Excessively high concentrations of Ca^2+^ change the structure of α-synuclein from soluble to insoluble, which forms aggregates and impairs synaptic transmission [5]. Similarly, in HD, mitochondrial function is also impaired [6]. In addition, mutant huntingtin has the potential to interact with other proteins such as calmodulin and calsequestrin, which can lead to a disruption in their ability to regulate Ca^2+^ levels [7]. Ca^2+^ also play a multifaceted role in the pathogenesis of migraine. Mutations in calcium channels are often at the root of migraine, causing an excessive influx of Ca^2+^ into nerve cells, resulting in excessive neuronal stimulation and triggering a migraine attack. The channelopathies are most often associated with hemiplegic migraine and involve mutations mainly in the *CACNA1A* gene encoding P/Q-type calcium channels, which leads to an excessive influx of Ca^2+^ into neurons and consequently contributes to the increased release of neurotransmitters and activation of the neuronal depolarization mechanism [8]. Ca^2+^ regulate the release of neuropeptides at synapses, including calcitonin gene-related peptide (CGRP). High ion concentrations lead to excessive release of CGRP, causing vasodilation in the meningeal area, resulting in mechanical pressure on nociceptors, which causes migraine pain [9]. Epilepsy is another of the diseases associated with abnormal calcium ion regulation. Some forms of epilepsy are associated with mutations in the *CACNA1H* gene, which encodes T-type calcium channels and results in a lowering of the threshold for activation of these channels and a prolongation of their opening time, which disrupts calcium homeostasis through an excessive influx of Ca^2+^ into neurons [10]. In the pathogenesis of this disease, there is a dysregulation of the balance between excitatory and inhibitory transmission. A high concentration of Ca^2+^ promotes the generation of an action potential by neurons, which leads to the release of more glutamate [11]. On the other hand, prolonged elevated calcium ion concentrations lead to desenthesis of GABAergic receptors, resulting in an impaired postsynaptic response even when gamma-aminobutyric acid (GABA) levels are normal [12].

This multifaceted role of Ca^2+^ in the pathogenesis of neurological diseases has allowed the development of treatments based on maintaining their homeostasis. Niomidipine, an L-type calcium channel blocker, may have applications in neurodegenerative diseases such as AD, PD, and HD due to its neuroprotective properties related to preventing excessive calcium ion influx into neurons [13,14,15]. Treatment with flunarizine and verapamil, which have the ability to affect the CaV2.1 calcium channel, has proven effective in some migraine patients [16,17]. Another promising therapeutic direction is calcium-binding proteins, such as parvalbumin, whose pharmacologically modulated activity can reduce the severity of epileptic seizures in some patients [18]. A detailed analysis of the role of Ca^2+^ in the physiological and pathological processes of the nervous system may contribute to a better understanding of the critical points in the pathogenesis of neurological diseases, which could potentially contribute to the development of increasingly effective targeted therapies related to the stabilization of Ca^2+^ levels.

## 2. The Role of Calcium Ions in the Functioning of the Central Nervous System

**Figure 1:**
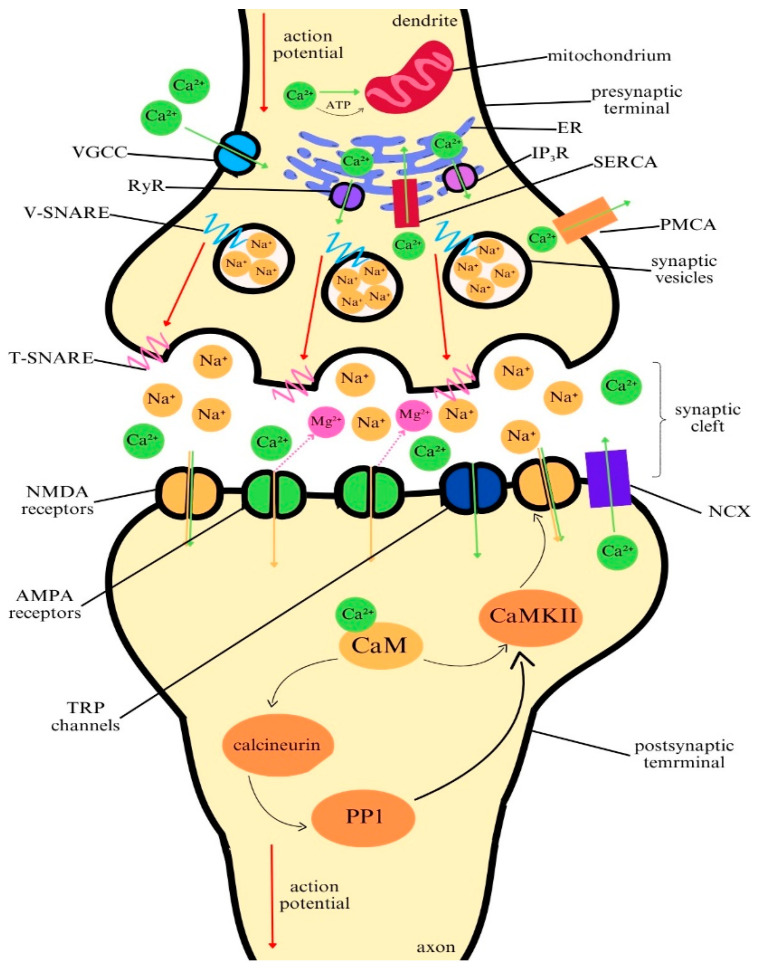
Calcium ions in the physiology of the central nervous system. Calcium plays a pivotal role in neural communication through regulating Ca^2+^ movement across the neuronal membrane. VGCCs are activated via action potential which triggers the fusion of vesicles with the presynaptic membrane. This process is facilitated by SNARE proteins. Ca^2+^ binds to calcium-binding proteins which are associated with long-term potentiation (LTP) and long-term depression (LTD). Calcium efflux occurs from the cells by TRP, PMCA, NCX and SERCA. VGCC: voltage-gated calcium channels; RyR: ryanodine receptors; NMDA: N-methyl-D-aspartate; AMPA: alpha-amino-3-hydroxy-5-methyl-4-isoxazolepropionic acid; TRP: transient receptor potential; ER: endoplasmic reticulum; IP3R: inositol trisphosphate receptor; SERCA: sarcoplasmic/endoplasmic reticulum calcium ATPase; PMCA: plasma membrane calcium ATPase; NCX: sodium–calcium exchanger; CaM: calmodulin; CaMKII: calcium/calmodulin-dependent protein kinase type II; PP1: protein phosphatase type I.

Ca^2+^ is crucial for properly functioning the central nervous system (CNS). It serves as a second messenger in various signaling pathways, regulates synaptic transmission, and modulates synaptic plasticity, which are crucial for neural communication, learning, and memory. The proper maintenance of calcium homeostasis is essential for normal neuronal function, and any dysregulation in its signaling can lead to significant alternations in neural processes (Figure 1) [19].

### 2.1. The Role of Calcium in Neuronal Communication

Neuronal communication is fundamentally dependent on the movement of ions across the neuronal membrane, and Ca^2+^ play a pivotal role in this mechanism. In response to action potentials, voltage-gated calcium channels (VGCCs) open, allowing Ca^2+^ to enter into the presynaptic terminal [20,21]. At the presynaptic terminal, VGCCs are activated by the incoming action potential. This phenomenon allows a rapid influx of Ca^2+^ into the presynaptic membranes, subsequently triggering the fusion of neurotransmitter-filled vesicles with the presynaptic membrane. The whole process is facilitated by SNARE proteins. V-SNAREs are associated with the vesicles, while T-SNAREs are placed on destined membrane [22]. This fusion induces the release of neurotransmitters into the synaptic cleft where they bind to receptors on the postsynaptic cell. This interaction affects the postsynaptic membrane potential, making Na^+^ channels open followed by the initiation of an action potential, and the continuation of the signal to the next neuron. The process of neurotransmitter release is highly sensitive to calcium levels, and the concentration of Ca^2+^ in the presynaptic terminal determines the strength and frequency of synaptic transmission. This makes Ca^2+^ not only essential for the transmission of electrical signals between neurons but also key regulators of synaptic activity and plasticity [20,23].

### 2.2. Calcium as a Second Messenger in Signal Transduction

Apart from its role in neurotransmitter release, calcium is commonly known as the second messenger in various intracellular signaling cascades, controlling all aspects of cellular functioning [24]. Ca^2+^ binds to a range of calcium-binding proteins, particularly calmodulins, which is followed by the activation of several downstream pathways. Hundreds of proteins contain calmodulin recruitment sites and therefore are susceptible to calcium control [25]. Once calmodulin is activated, it acts as a “molecular switch”, interacting with and activating a wide range of proteins. These include kinases, phosphatases, ion channels, and other enzymes that regulate cellular functions [25,26]. For instance, calcium/calmodulin-dependent protein kinases (CaMKs, highly enriched in the brain, particularly in the hippocampus) are crucial for a process that is essential for synaptic plasticity, which improves or weakens synaptic connections in response to activity, thus underpinning memory formation and learning [27]. This mechanism is strongly associated with long-term potentiation (LTP) and long-term depression (LTD). Both LTP and LTD are forms of activity-dependent synaptic modification, but they lead to opposite outcomes in terms of synaptic strength: LTP increases it, while LTD decreases it [28,29]. LTP is typically triggered by the activation of N-methyl-D-aspartate (NMDA) receptors, which are located on the postsynaptic membrane and are sensitive to glutamate [30]. NMDA receptors have a unique property: they are both ligand-gated and voltage-dependent. This means that they require two conditions to open, namely glutamate binding and membrane depolarization. Once Ca^2+^ enters the postsynaptic neuron through NMDA receptors, it initiates a cascade of intracellular signaling events. The result of the number and efficiency of α-amino-3-hydroxy-5-methyl-4-isoxazolepropionic acid (AMPA) receptors increases, making the synapse more responsive to future synaptic inputs. The synapse becomes “potentiated”, meaning that it responds more robustly to the same level of presynaptic stimulation, which is the known hallmark of LTP [28,30,31]. In contrast to LTP, LTD weakens synaptic connections, leading to a reduction in synaptic strength. LTD is also a calcium-dependent process but requires lower, prolonged, and more sustained exposure to calcium signals than LTP. Similarly, LTD in many brain regions, such as the hippocampus and cerebellum, is initiated by calcium influx through NMDA receptors [29,30]. The reduced levels of calcium that enter the postsynaptic neuron in LTD preferentially induce a different set of signaling pathways. For instance, lower calcium concentrations favor the activation of calcineurin, a calcium/calmodulin-dependent phosphatase, affecting protein phosphatase 1 (PP1) activity. Once activated by calcineurin, PP1 further dephosphorylates proteins that are essential for maintaining high synaptic efficacy, such as AMPA receptors [32]. This leads to the internalization or removal of AMPA receptors from the postsynaptic membrane, reducing synaptic sensitivity to glutamate. This reduction in receptor density diminishes the responsiveness of the synapse to presynaptic signals, resulting in weakened synaptic transmission. LTD helps in refining neural circuits by weakening unnecessary or redundant synapses [29,30,31,33].

### 2.3. Calcium Homeostasis in Neurons and Astrocytes

Calcium homeostasis in neurons is a fundamental aspect of cellular function, ensuring that intracellular Ca^2+^ levels are precisely regulated to support several physiological processes. Because prolonged or excessive calcium influx can lead to cellular damage, maintaining calcium concentration within narrow limits is critical for neuronal health and survival. Neurons manage calcium homeostasis through several mechanisms. As mentioned, it enters the cell from the extracellular space through VGCCs during action potentials, facilitating neurotransmitter release and activating synaptic plasticity pathways. Additionally, NMDA receptors allow calcium influx when induced by glutamate [29,30]. Another important entry route involves transient receptor potential (TRP) channels which are activated by different external stimuli, including temperature changes (such as heat activating TRPV1 or cold triggering TRPM8), mechanical stress (detected by TRPA1 and TRPC1), and chemical signals (like capsaicin activating TRPV1 or irritants activating TRPA1). Additionally, TRPM2 channels respond to oxidative stress, helping regulate calcium levels during cellular stress [34].

The endoplasmic reticulum (ER) is the main reservoir for calcium within neurons. Calcium is released from the ER through inositol 1,4,5-trisphosphate (IP_3_) receptors, which are activated by signaling pathways triggered by neurotransmitters, i.e., glutamate or acetylcholine. When IP_3_ binds to its receptor on the ER membrane, it triggers calcium release into the cytoplasm. Another pathway includes ryanodine receptors (RyR), also incorporated in the ER membrane. These proteins take part in the process called calcium-induced calcium release (CICR). Namely, when a small amount of calcium enters the cell from the outside (e.g., VGCCs), this calcium can bind to RyR, triggering an even larger influx of Ca^2+^ [21,24,35].

Mitochondria, though not the main storage site for calcium, are involved in the regulation of calcium levels during periods of high neuronal activity. When neurons are highly active, calcium levels in the cytoplasm rise. Mitochondria temporarily take up this excess through the mitochondrial calcium uniporter (MCU) to prevent calcium overload. By performing this specific kind of buffering, mitochondria help maintain intracellular balance. The calcium uptake is also associated with the energy metabolism, as it stimulates the production of ATP, which is essential for supporting the cell’s increased energy requirements during activity. Once the activity diminishes, mitochondria gradually release the sequestered calcium back into the cytoplasm or other storage sites [19,24,35,36,37].

To prevent calcium overload, neurons utilize buffering systems that maintain calcium within safe limits. A range of calcium-binding proteins, such as calbindin, parvalbumin, and calretinin, rapidly bind to free calcium ions, preventing harmful fluctuations in calcium concentration [1]. Additionally, several calcium pumps actively remove excess calcium from the cytoplasm. The plasma membrane calcium ATPase (PMCA) uses ATP to pump calcium out of the cell, maintaining low resting levels, while the sodium–calcium exchanger (NCX) uses the sodium gradient to expel calcium following synaptic transmission or action potentials [25]. Inside the cell, the sarco/endoplasmic reticulum calcium ATPase (SERCA) pump replenishes ER calcium stores after release, maintaining proper intracellular calcium balance [38,39].

While much of the focus has been on the role of calcium in neurons, glial cells—particularly astrocytes—also rely heavily on calcium signaling to fulfill their supportive and regulatory roles in the CNS. Astrocytes respond to neurotransmitter release by increasing intracellular calcium levels, which then triggers the release of gliotransmitters such as ATP and glutamate. These gliotransmitters can modulate synaptic transmission and contribute to the regulation of neuronal excitability.

Astrocytes are also involved in the maintenance of extracellular calcium levels, preventing excessive accumulation of Ca^2+^ in the synaptic cleft and thereby protecting neurons from potential excitotoxicity [40].

## 3. Channelopathies Related to Impaired Transport of Calcium Ions in Neurology

Calcium channels play a key role in the functioning of the nervous system, regulating, among others, neuronal depolarization, neurotransmitter release, and various intracellular signaling processes [41]. Calcium channels consist of alpha-1, beta, alpha-2/delta, and gamma subunits. The alpha-1 subunit, which forms the channel pore, regulates channel activity, while the remaining subunits act as auxiliary elements [42]. Channelopathies are a group of diseases resulting from mutations in the genes encoding ion channel subunits, which leads to the incorrect transportation of ions across cell membranes [43]. Disturbed ion transport can affect various types of ions, including sodium, potassium, chlorine, and calcium. Because calcium is an important intracellular signaling molecule, altered calcium channel function can cause widespread changes in neuronal function [44]. Currently, more and more attention is being paid to the importance of channelopathy in the pathophysiology of neurological diseases, such as migraine [45].

Ion channel genetic variants are frequently classified as either a loss of function (LOF) or a gain of function (GOF). This LOF/GOF classification is often directly used to predict the effects on neuronal firing, which in turn is important for understanding the pathophysiology of these disorders and for the identification of potential therapeutic targets [46].

### 3.1. CACNA Gene Family-Related Channelopathies

VGCC are important for controlling the influx of calcium into cells in response to changes in membrane potential, especially in the nervous system, where they are responsible for the release of neurotransmitters (e.g., glutamate) at presynaptic terminals [47].

The *CACNA* gene family, which encodes the alpha subunits of the VGCC complexes, is responsible for the formation of functional calcium channels. These genes are located on different chromosomes and show specific expression in various tissues, with particular emphasis on the nervous system [48,49]. The activity of *CACNA* genes and their protein products is tightly regulated at many levels to ensure precise control of calcium influx and neuronal excitability. Regulation can occur through a variety of mechanisms, including post-translational modifications, alternative splicing, and protein–protein interactions [49].

#### 3.1.1. *CACNA1A* Gene

The *CACNA1A* (calcium voltage-gated channel subunit alpha1 A) gene, located on chromosome 19p13.1, encodes the alpha1 A subunit of the P/Q-type calcium channel, also known as the Cav2.1 subunit, located mainly in the presynaptic regions of the cerebral cortex, thalamus, hypothalamus, hippocampus, and cerebellum [50]. The *CACNA1A* mutations result in a wide spectrum of consequences, including increased probability of channel opening, lower activation voltage leading to increased Ca^2+^ influx, reduced channel inhibition by G protein βγ heterodimers, altered synaptic morphology, excitation–inhibition imbalance, and increased glutamate release [8,51]. Gain-of-function mutations in Cav2.1 contribute to the development of familial hemiplegic migraine type 1 (FHM1) [52]. Of the more than 25 different genetic variants identified in the FHM1-related *CACNA1A* gene, missense variants such as T666M, R192Q, and S218L are the most common [49,53]. The T666M variant involves substituting threonine with methionine at position 666 in the L-type calcium channel subunit protein sequence. Similarly, the R192Q variant involves replacing the amino acid arginine with glutamine at position 192 in the protein sequence, while the S218L variant leads to the substitution of serine at position 218 with leucine [54,55].

#### 3.1.2. *CACNA1B* Gene

The *CACNA1B* (calcium voltage-gated channel subunit alpha1 B) gene, located on chromosome 9q34.3, encodes the alpha1 B subunit of the VGCC channel, also known as the Cav2.2 subunit [56], found primarily in presynaptic regions of the midbrain, cerebellar cells, spinal cord motoneurons, and cholecystokinin-expressing interneurons [57]. In 2015, Groen et al. [58] described a mutation of the *CACNA1B* gene, R1389H (c.4166G>A; p.Arg1389His) in dystonia. This mutation is a disruptive missense mutation in the outer region of the ion pore. The c.4166G>A variant leads to a reduction in the ion current flowing through the Cav2.2 channel, thereby affecting the release of neurotransmitters at inhibitory and excitatory synapses.

#### 3.1.3. Other *CACNA* Family Genes

Mutations in other *CACNA* family genes also play an important role in the pathophysiology of neurological diseases. For example, the *CACNA1C* (calcium voltage-gated channel subunit alpha1 C) gene, located on chromosome 12p13.33, encodes the alpha-1C subunit of L-type calcium channels, which play a key role in cardiac and neuronal excitability, muscle contraction, and synaptic plasticity [59,60]. The rs1006737 *CACNA1C* genetic variant has been associated with altered amygdala function, intellectual disability, autism, developmental disorders and cardiac arrhythmias [61]. The *CACNA1E* (calcium voltage-gated channel subunit alpha1 E) gene encodes the alpha-1E subunit (Cav2.3) of the voltage-gated calcium channel. At least 14 missense mutations have been identified in this gene, of which approximately 60% of cases involve three genetic variants: c.1054G>A (p.Gly352Arg), c.2104G>A (p.Ala702Thr) and c.2101A>G (p.Ile701Val). These changes occur at the cytoplasmic ends of all S6 transmembrane segments that form the activation gate, resulting in observed gain-of-function effects. In cases where genetic changes result in loss-of-function, a milder clinical phenotype is observed [49,62].

### 3.2. TRP Gene Family-Related Channelopathies

Transient receptor potential (TRP) channels are a conserved family of Ca^2+^-permeable cation ion channels that are widely expressed in the brain—both neurons and glial cells—and in cerebrovascular endothelium and smooth muscle [63]. TRP channels are classified into six main families: TRPA, TRPC, TRPM, TRPP, TRPL and TRPV [64]. The influx of cations through TRP channels leads to depolarization of the neuronal membrane potential and an increase in intracellular Ca^2+^ concentration, which plays an important role in the regulation of many cellular processes [57].

In recent years, the *TRPA1* (Transient Receptor Potential Ankyrin 1) gene has attracted particular interest in the context of its involvement in the pathogenesis of neurological diseases. The TRPA1 protein plays a key role in the transmission of pain signals and sensitivity to cold, and its mutations correlate with the occurrence of various neurological diseases, including chronic pain [65,66]. For example, the c.3053A>G polymorphism in the *TRPA1* gene has been identified as a factor influencing migraine in young age [66]. Moreover, the homozygous GG genotype of this c.3053A>G variant is associated with increased pain response. In silico analyses suggest that this benign variant may affect protein structure and splice site, which could potentially modulate TRPA1 channel function [67].

Conducting detailed functional studies on the mutations related to calcium channels genes could enhance our understanding of their specific contributions to neuronal excitability and synaptic transmission.

## 4. The Role of Calcium Ions in Pathology Central Nervous System

### 4.1. Calcium Ions in Pathology of Alzheimer’s Disease

**Figure 2:**
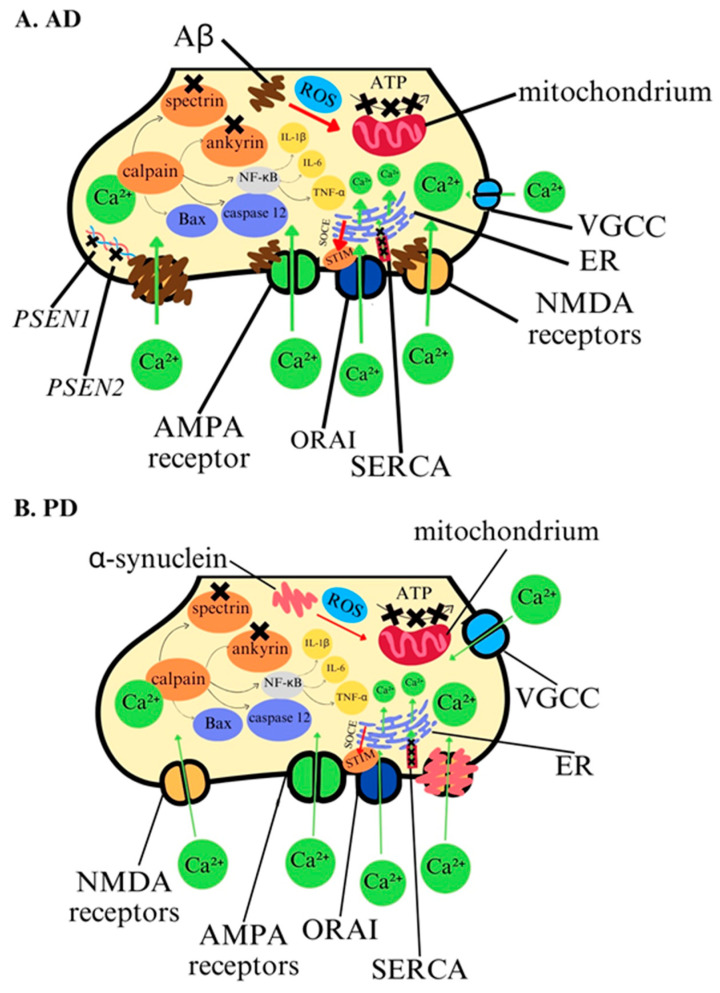
Calcium ions in the pathology of AD and PD. (**A**). In AD, disruption occurs in Ca^2+^ signaling between ER and mitochondria and Ca^2+^ efflux impairment due to dysfunction of SERCA. Excessive calcium influx promotes Aβ aggregation and is triggering apoptotic pathways. (**B**) Ca^2+^ dysregulation in PD is similar to AD. Excessive Ca^2+^ influx leads to activation of apoptotic pathways and promotes α-synuclein aggregation. Under physiological conditions, SERCA efficiently transports Ca^2+^ from the cytoplasm to the ER, which prevents calcium accumulation in the cell. Mitochondria play a key role in regulating Ca^2+^ levels and ATP production. These processes can be disrupted by abnormal amyloid beta and α-synuclein proteins.

Ca^2+^ play a crucial role in neuronal function, and their dysregulation is key feature of AD pathology (Figure 2A, Table 1). Disruptions in calcium signaling between the endoplasmic reticulum (ER) and mitochondria lead to mitochondrial dysfunction, oxidative stress, and neuronal degeneration. In AD, Aβ exacerbates calcium influx into neurons, triggering apoptotic pathways like the activation of calpain, a calcium-dependent protease, which degrades essential proteins and promotes brain inflammation. Prolonged Ca^2+^ elevation also results in tau hyperphosphorylation, worsening synaptic dysfunction. Ca^2+^ modulates Aβ aggregation, promoting toxic Aβ oligomer formation. Impaired calcium efflux from the ER and mitochondria heightens neurons’ vulnerability to apoptosis [68,69]. Ca^2+^ is essential for synaptic plasticity, but in AD, these processes are disrupted. Mutations in presenilin, *PSEN1* and *PSEN2* genes increase Ca^2+^ release from the ER, causing synaptic dysfunction [70,71]. This disruption, combined with dendritic and spine loss observed in AD patients, correlates with cognitive decline [72,73]. Postsynaptically, Aβ oligomers interact with NMDARs and VGCCs, leading to excitotoxicity and neuronal death, particularly in memory-associated brain regions like the hippocampus [74,75]. Calcium-permeable AMPA receptors further contribute to this neurotoxicity [76]. Therapeutic strategies targeting calcium dysregulation are promising. Calcium channel blockers like isradipine mitigate Aβ-induced calcium influx, providing neuroprotection [77]. Modulating VGCC and TRP channels, particularly TRPC6, may prevent calcium-induced excitotoxicity and synaptic failure [78,79]. Overall, Ca^2+^ dysregulation is a core mechanism in AD, and targeting these disruptions offers potential therapeutic pathways to slow or halt disease progression.

### 4.2. Calcium Ions in Pathology of Parkinson’s Disease

In PD, Ca^2+^ dysregulation plays a key role in dopaminergic neuron degeneration (Figure 2B, Table 1). Excessive calcium influx through L-type calcium channels triggers apoptotic processes, contributing to neuronal death. Elevated intracellular Ca^2+^ also promotes α-synuclein aggregation, a hallmark of PD. Ca^2+^ gradients, essential for cellular signaling, regulate processes such as excitability, neurotransmitter release, and apoptosis [80,81]. The activity of calcium ions is intrinsically associated with synaptic plasticity in PD, particularly through their critical role in dopaminergic transmission. Calcium influx via voltage-gated calcium channels in dopaminergic neurons regulates dopamine release, which is essential for maintaining motor and cognitive functions. Dysregulation of calcium dynamics disrupts mechanisms such as LTP and LTD, which are fundamental processes of synaptic plasticity. These impairments contribute to the progressive degeneration of dopaminergic pathways, culminating in motor dysfunction and other hallmark features of PD [82,83]. In the substantia nigra, defects in calcium homeostasis accelerate neurodegeneration, with PD patients showing higher calcium levels than healthy individuals [84]. Calcium enters dopaminergic neurons via VGCCs, NMDA, and AMPA receptors, and is released from the ER and mitochondria, with intracellular levels tightly controlled by various pathways [85]. Therapeutic approaches aim to modulate Ca^2+^ signaling to reduce neuronal loss and improve motor function. Calcium channels blockers have shown potential in slowing dopaminergic neurodegeneration [86,87], with DHP-type calcium channel blockers reducing PD risk by around 25% [88,89]. In PD, α-synuclein disrupts Ca^2+^ delivery from the ER to mitochondria, disturbing calcium homeostasis [90,91]. Understanding these mechanisms is crucial for developing therapies that maintain calcium balance and prevent neurodegeneration.

### 4.3. Calcium Ions in Pathology of Huntington’s Disease

**Figure 3:**
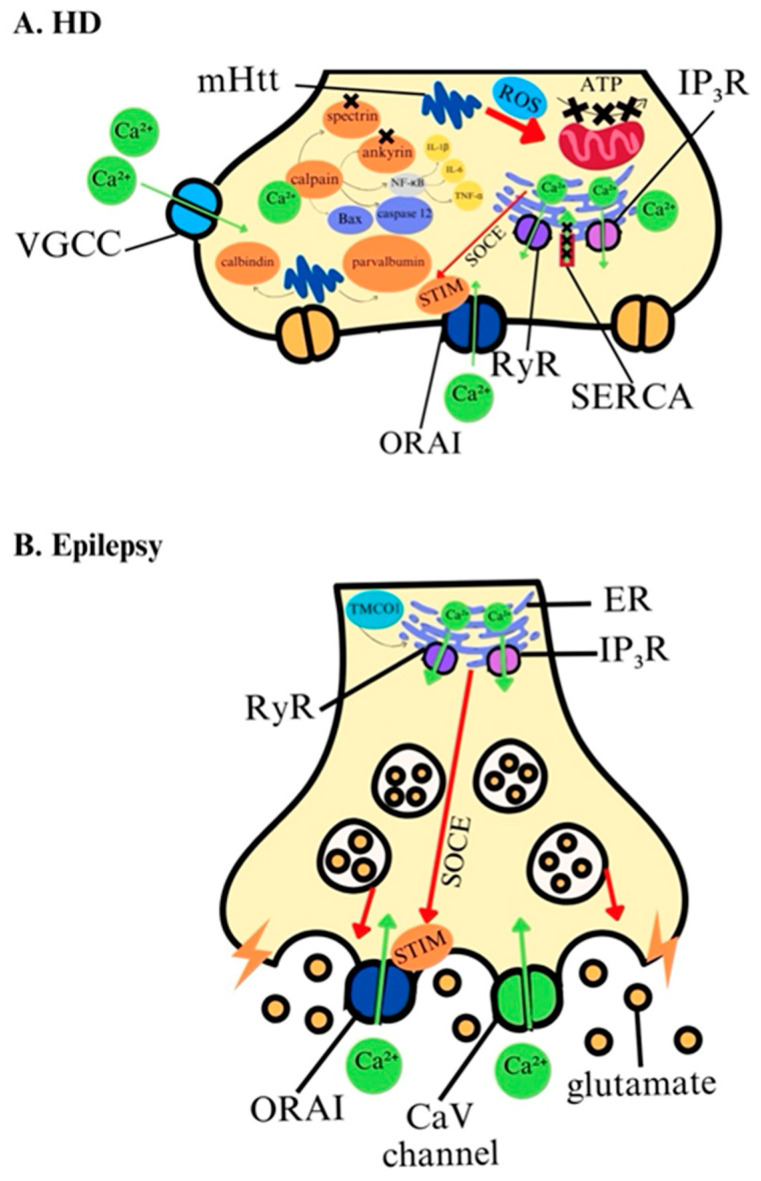
Calcium ions in the pathology of HD and epilepsy. (**A**) In HD, calcium signaling is disrupted due to mHtt interactions with calcium-binding proteins mitochondria and SERCA. mHtt promotes ER Ca^2+^ release which leads to abnormal activation of SOCE. (**B**) In epilepsy, elevated Ca^2+^ levels result in synchronized neuronal firing which contributes to seizure activity. Excessive calcium release from ER leads to abnormal activation of SOCE. Under physiological conditions, SOCE allows for the replenishment of Ca^2+^ in the ER, but its excessive activity leads to calcium overload.

In HD, disrupted calcium signaling is a central feature linked to mutant huntingtin (mHtt) results from an expanded CAG repeat in the *HTT* gene, leading to calcium imbalance and mitochondrial dysfunction (Figure 3A, Table 1). Neuronal calcium signaling depends on proteins that regulate free Ca^2+^ levels, ATPase, and Na^+^/Ca^2+^ exchangers, along with calcium channels [92,93]. mHtt interacts directly with calcium-binding proteins, increasing intracellular Ca^2+^ and impairing protein function, including calmodulin, whose disruption affects neuroprotection [94,95,96]. Elevated Ca^2+^ level activates calpain, a protease that degrades cellular proteins, accelerating neurodegeneration. Inhibiting calpain in Drosophila HD models prevents mHtt toxicity by enhancing autophagy, while calpain inhibitor calpastatin increases autophagosome formation, showing therapeutic potential [97]. At the transcriptional level, mHtt fragments alter calcium-regulating gene expression in HD models, including genes for calcium-binding proteins and calcium channels [98,99,100]. Reduced expression of the SERCA2 calcium pump has been observed in HD [101]. mHtt also enhances ER calcium release through interaction with InsP3R1, leading to store depletion and abnormal activation of store-operated calcium entry (SOCE), a process also disrupted in other neurodegenerative diseases [102,103,104]. Increased SOCE amplitude in HD models suggests these disturbances are key in disease progression [105,106]. Mitochondrial Ca^2+^ dysregulation further contributes to HD, where impaired ER–mitochondria calcium transfer increases mitochondrial fragmentation and dysfunction, worsening neurodegeneration. Mitochondria-targeted therapies, such as mitochondrial permeability transition inhibitors, have shown neuroprotective effects in HD models [107,108,109,110,111].

### 4.4. Calcium Ions in Epilepsy

In epilepsy, Ca^2+^ homeostasis dysregulation significantly contributes to neuronal hyperexcitability and seizure generation (Figure 3B, Table 1). Calcium channels, especially T-type and L-type, are crucial for action potential propagation and neurotransmitter release. The ER releases Ca^2+^ via inositol trisphosphate receptors (IP3Rs) and ryanodine receptors (RyRs) to elevate cytosolic calcium levels [112]. Dysregulated Ca^2+^ signaling can lead to excessive glutamate release, enhancing excitotoxicity and seizure susceptibility. Ca^2+^ is a vital intracellular messenger for neurotransmitter release, and its disruption can result in neurological disorders like epilepsy [112,113,114,115]. Elevated intracellular calcium promotes synchronized neuronal firing, contributing to seizure activity. Key components of calcium homeostasis include CaV channels, which comprise a pore-forming α1 subunit and auxiliary β and α2δ subunits [116]. The main CaV groups—CaV1, CaV2, and CaV3—serve distinct roles, with CaV2 facilitating Ca^2+^ entry in presynaptic terminals [117]. SOCE functions independently of CaV channels and is activated by calcium depletion in the ER, where the calcium sensor STIM interacts with the PM calcium channel Orai [118,119]. The ER also experiences passive leakage and active Ca^2+^ release, influenced by proteins like TMCO1 and various modulators [120]. NCXs are crucial for calcium clearance, maintaining low cytosolic levels [121,122]. Genetic mutations in genes encoding CaV channels, including CaV1.2, CaV1.3, CaV2.1, CaV2.2, CaV2.3, and CaV3.2 [123,124,125,126,127,128,129,130,131], highlight the importance of calcium dysregulation in seizures.

### 4.5. Calcium Ions in Migraine

**Figure 4:**
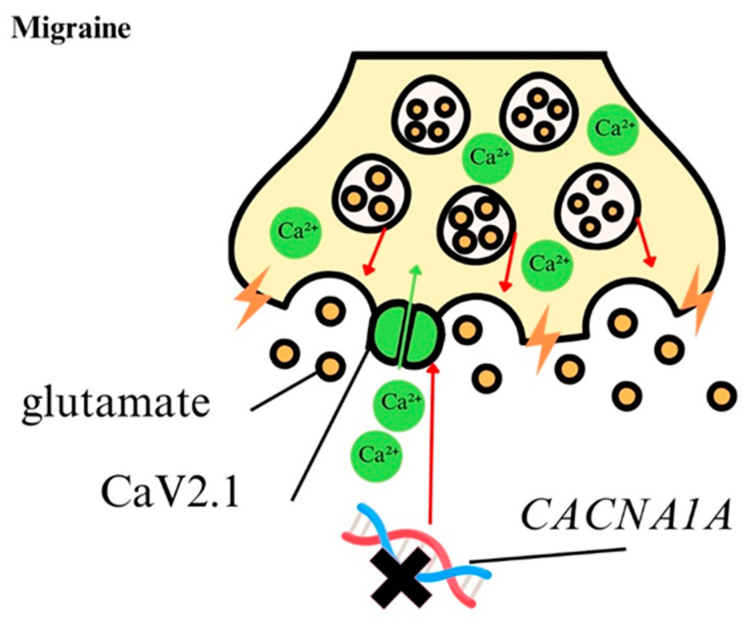
Calcium ions in the pathology of migraine. In migraine, there is a dysfunction of ion channels resulting in elevated Ca^2+^ levels. Some genetic variants of the *CACNA1A* gene lead to excessive calcium influx and excitatory synaptic transmission, which contribute to migraine pathogenesis. Under physiological conditions, ion channels precisely regulate the influx of Ca^2+^ into the cell, which supports the maintenance of calcium homeostasis.

Migraine is a prevalent neurological condition with significant public health implications. Due to its complex, multifactorial, and polygenic pathophysiology, the precise mechanisms underlying migraine remain poorly understood (Figure 4, Table 1). The cortical spreading depression (CSD) hypothesis suggests that migraines may arise from ion channel dysfunction, leading to elevated levels of Ca^2+^ and increased glutamate release in synaptic clefts, which in turn trigger CSD through presynaptic P/Q-type Ca^2+^ channels [8,132,133,134,135]. The *CACNA* gene family, particularly *CACNA1A*, plays a key role in a rare subtype of migraine characterized by motor aura and heightened synaptic activity, driven by increased CaV2.1 channel function, ultimately resulting in CSD [136,137]. Genetic variants of *CACNA1A* have been shown to increase calcium influx and excitatory synaptic transmission, contributing to migraine pathogenesis [54,55,136,137,138,139,140,141,142]. Additionally, other genes correlated to Ca^2+^ within the *CACNA* family, such as *CACNA1B* and *CACNA1E*, have been studied for their potential role in migraine development [143,144,145,146,147].

**Table 1:** The role of calcium ions in pathology of neurological disorders.

Neurological Disease	Biochemical Changes	Cellular Changes	Genetic Factors	Clinical Symptoms	Diagnostic/ Therapeutic Factor	References
Alzheimer’s disease (AD)	Calpain activation, Calcium-permeable receptors; AMPA NMDA VGCC, apoptosis induction.	Mitochondrial and endoplasmic reticulum dysfunction, oxidative stress generation, neuronal degeneration, beta-amyloid aggregation, excitotoxicity.	Mutation in AD-causal genes; *PSEN1* *PSEN2* leading to amyloid cascade.	Cognitive decline.	Calcium ion antagonists-dihydropyridine to inhibit calcium overload, calcium ion inhibitor-curcumin, restoring mitochondrial calcium homeostasis-aduhelm?	[68,69] [72,73,74,75,76,77,78,79] [148,149,150]
Parkinson’s disease (PD)	Calcium-permeable receptors; AMPA NMDA VGCC, calpain activation, apoptosis induction.	Alpha-synuclein aggregation, Mitochondrial and endoplasmic reticulum dysfunction, oxidative stress generation, dopaminergic neurons degeneration in the substantia nigra. excitotoxicity.	Mutation in PARK genes leading to Lewy bodies aggregation.	Motor dysfunction.	Calcium channel blockers- Isradipine.	[80,81,82,83] [151,152] [153,154,155,156]
Huntington’s disease (HD)	Calcium-permeable receptors; AMPA NMDA VGCC, calpain activation, apoptosis induction, store-operated calcium entry (SOCE) mechanism activation.	Mutant huntingtin (mHtt) interacts directly with calcium-binding proteins calbindin and parvalbumin, Mitochondrial and endoplasmic reticulum dysfunction, oxidative stress generation.	Mutation in *HTT* gene, expanded CAG repeat.	Motor dysfunction, cognitive decline.	Compound stabilizing SOCE-tetrahydro-carbazoles as candidate for targeted HD therapy, potential targets for maintaining calcium homeostasis- RyR, IP3R.	[92,93,94,95,96,97,98,99,100,101,102,103,104,105,106,107,108] [157,158,159,160]
Epilepsy	Store-operated calcium entry (SOCE) mechanism activation, excessive glutamate release, excitotoxicity	Endoplasmic reticulum dysfunction, promotes synchronized neuronal firing.	Channelopathies of CaV subunits.	Seizure susceptibility.	Potential targets for preventing epileptic seizures-CaBPs like calmodulin, parvalbumin, calretinin, calbindin.	[113,114,115,116,117,118,119,120,121] [161,162,163,164,165,166,167,168,169]
Migraine	Excessive glutamate release, excitotoxicity.	Cortical spreading depression (CSD) genering.	Channelopathies of CaV subunits.	Migraine attack.	Calcium channels blockers- flunarizine, verapamil, inhibitor of ASIC1a- amiloride, tarantula toxin.	[8,133,134,135,136] [144,145,146,147] [170,171,172,173,174]

## 5. Potential Role of Calcium Ions in Diagnostics and Therapy of Neurological Diseases

### 5.1. Calcium Ions as Diagnostic and Therapeutic Target in Alzheimer’s Disease

Ca^2+^ play a crucial role in the diagnostics and therapy of AD, underscoring their significance in both understanding the disease’s pathology and developing treatment strategies (Table 1). The relationship between Ca^2+^ homeostasis and AD is well established, as disturbances in calcium signaling are observed during the early stages of the disease, which may influence cognitive decline and other clinical outcomes [175]. Research supports the idea that targeting calcium homeostasis is a feasible approach for treating AD, with Ca^2+^ antagonists serving as the primary type of clinically developed therapeutic drugs aimed at regulating these ions. For instance, Ca^2+^ antagonists, particularly dihydropyridine drugs like nimodipine, aim to inhibit calcium overload; however, clinical trials have shown that these agents do not significantly reduce the rate of cognitive decline in patients with AD [148]. In addition to Ca^2+^ antagonists, other compounds are being explored for their ability to modulate calcium homeostasis. Curcumin, an intracellular calcium inhibitor, has demonstrated neuroprotective effects by attenuating glutamate-induced neurotoxicity, protecting astrocytes from oxidative stress, and inhibiting apoptosis in experimental models [149]. More recently, Aduhelm, a new drug approved by the FDA, not only targets amyloid plaques but also has the potential to ameliorate calcium overload, as suggested by prior studies [150]. This multifaceted action could make it a promising candidate for more effective AD therapies. Moreover, interventions aimed at restoring mitochondrial calcium homeostasis are gaining traction as potential therapeutic strategies for AD. The MCU is a key player in regulating mitochondrial Ca^2+^ levels, and its inhibition may provide a novel target for improving cognitive function in AD patients [176,177]. For example, TG-2112x, a compound that partially inhibits mitochondrial calcium uptake, has shown promise in protecting neurons in preclinical studies [178]. Additionally, recent studies propose that inhibiting the mitochondrial permeability transition pore (mPTP) may ameliorate cognitive deficiencies in AD transgenic mice, suggesting a critical link between mitochondrial function and calcium dysregulation [179]. Restoring intracellular calcium homeostasis by modulating receptors on the endoplasmic reticulum, particularly ryanodine receptors (RyRs), represents another area of active research. Enhanced Ca^2+^ leakage through RyR channels can activate altered calcium-dependent signaling pathways seen in AD [180]. Therapeutics like Rycal S107 have shown potential in repairing Ca^2+^ leakage and reducing neuropathological changes without significant side effects [181]. Emerging evidence also suggests that cannabinoid-based therapies may modulate RyRs, potentially reversing AD-related pathological changes and cognitive deficits [182]. As the understanding of calcium’s multifaceted role in AD deepens, it becomes apparent that developing novel interventions focused on calcium homeostasis is vital for improving diagnosis and treatment. The various pathways linking calcium dysregulation with AD manifestations warrant further investigation to discover more effective targeted therapies. Thus, they not only serve as critical biomarkers for early detection of AD but also represent promising therapeutic targets that could lead to improved outcomes for patients [183]. In summary, ongoing research into the intricate relationship between calcium signaling and AD pathology is essential for advancing therapeutic strategies aimed at mitigating the impact of this debilitating disease. Future research should prioritize the exploration of novel agents and combinational therapies targeting calcium homeostasis to provide enhanced efficacy in the treatment of Alzheimer’s disease. Substances such as magnesium-L-threonate, which has shown potential in improving synaptic density and cognitive functions through its impact on calcium signaling, warrant further investigation [184]. Additionally, targeting the SOCE pathway using inhibitors like SKF-96365 or YM-58483 could provide insights into the mitigation of calcium overload and its downstream neurotoxic effects [185,186]. Lastly, the application of advanced drug delivery systems, such as nanoparticles designed to cross the blood–brain barrier, could enhance the effectiveness of these interventions, marking a promising direction for future therapeutic advancements.

### 5.2. Calcium Ions as Diagnostic and Therapeutic Target in Parkinson’s Disease

Ca^2+^ play a crucial role in both the pathogenesis and potential treatment of PD, with their dysregulation contributing significantly to neuronal degeneration (Table 1). Normal calcium signaling is essential for various cellular functions in the brain, but an overload of Ca^2+^can lead to excitotoxicity, particularly damaging dopaminergic neurons in the substantia nigra pars compacta (SNPc) [151,152]. This calcium overload is linked to oxidative stress and mitochondrial dysfunction, processes intimately involved in PD’s etiology, as they exacerbate neuronal injury through mechanisms like increased oxidative stress and harmful free radical production. Research indicates that targeting calcium channels, specifically L-type calcium channels (LTCCs), may offer therapeutic avenues; calcium channel blockers (CCBs), such as isradipine, have shown promise in preventing excitotoxicity and protecting dopaminergic neurons [187,188]. Epidemiological studies support the idea that individuals using dihydropyridine CCBs experience a lower incidence of PD compared to other antihypertensive medications [153,154]. Overall, the interplay between calcium signaling and neurodegeneration in PD suggests that Ca^2+^ could be integral to both diagnostic strategies and the development of therapies aimed at mitigating disease progression. Future research should focus on developing therapies targeting calcium dysregulation in PD, leveraging both pharmacological and advanced molecular techniques. Novel LTCC inhibitors, such as nilvadipine, offer promise in reducing neurodegeneration beyond isradipine and warrant further investigation in preclinical and clinical settings [155]. Additionally, mitochondrial calcium regulation is an emerging field, with potential targets such as the MCU. Compounds like CGP-37157, an inhibitor of the mitochondrial sodium–calcium exchanger, may provide neuroprotection by mitigating mitochondrial calcium overload and reducing oxidative stress [156,189]. Finally, nanotechnology-based drug delivery systems designed to target specific calcium dysregulation pathways and improve the bioavailability of existing therapies represent a promising frontier. Collaborative efforts in these areas are critical to developing novel, targeted, and effective therapies for PD.

### 5.3. Calcium Ions as Diagnostic and Therapeutic Target in Huntington’s Disease

Ca^2+^ play a crucial role in both the pathogenesis and potential therapeutic approaches for HD, particularly through their involvement in calcium signaling dysregulation (Table 1). Abnormalities in SOCE0, which is essential for maintaining calcium homeostasis, have been linked to neurodegeneration in HD. In HD models, elevated synaptic SOCE has been observed in medium spiny neurons (MSNs), especially in YAC128 mice, and is thought to contribute to the loss of dendritic spines, a hallmark of HD-related neuronal damage [157,190]. This disruption in calcium homeostasis may accelerate neurodegeneration and contribute to early neuropathological changes, such as impaired corticostriatal synaptic function [191,192].

Therapeutic strategies focusing on stabilizing SOCE have shown promise in preclinical HD models. For instance, the specific SOCE inhibitor EVP4593 has been shown to normalize motor behavior and reduce synaptic spine loss in YAC128 mice by decreasing abnormal calcium influx [157,158]. Another potential treatment, tetrahydrocarbazoles, has demonstrated the ability to restore calcium homeostasis and stabilize SOCE in HD models, showing protective effects on mitochondrial function and neuronal survival [159]. These compounds selectively attenuate SOCE in HD-affected neurons without affecting normal ones, making them promising candidates for targeted HD therapy. In addition to small-molecule inhibitors, targeting intracellular calcium release channels, such as inositol 1,4,5-trisphosphate receptors (IP3Rs) and ryanodine receptors (RyRs), has also emerged as a therapeutic avenue. Dysregulated IP3R1 activity contributes to excessive calcium release from the endoplasmic reticulum (ER), exacerbating SOCE dysfunction in HD neurons. Interventions such as antisense oligonucleotides (ASOs) targeting IP3R1 or the use of specific IP3R1 blockers have shown potential in rescuing dendritic spine loss and normalizing calcium signaling [157,160]. Similarly, RyR inhibitors like dantrolene have been found to protect MSNs from glutamate-induced apoptosis, reduce motor deficiencies, and mitigate neuronal death in HD models [193,194]. These findings underscore the critical role of Ca^2+^ regulation in both the diagnosis and treatment of HD, highlighting calcium signaling pathways as key therapeutic targets for slowing disease progression. Future research should focus on developing therapies targeting calcium dysregulation in HD. Specific inhibitors of SOCE, like EVP4593, show promise and warrant further clinical investigation to reduce neurodegeneration. Additionally, targeting intracellular calcium release channels, such as IP3R1 and RyRs, may provide therapeutic benefits, with compounds like IP3R1 blockers and dantrolene demonstrating potential in preclinical models [195]. Mitochondrial calcium regulation is also a promising area, with compounds like CGP-37157 that mitigate mitochondrial overload and oxidative stress showing neuroprotective effects [196,197]. Moreover, nanotechnology-based drug delivery systems could enhance the precision and efficacy of these therapies. Collaborative efforts in these areas are essential for developing effective, targeted treatments for HD.

### 5.4. Calcium Ions as Diagnostic and Therapeutic Target in Epilepsy

Ca^2+^ play a central role in both the diagnosis and therapy of epilepsy due to their critical involvement in neuronal excitability and intracellular signaling (Table 1). Dysregulation of calcium channels has been directly linked to the initiation and propagation of epileptic seizures, primarily through abnormal increases in cytosolic calcium levels. Calcium-binding proteins (CaBPs), such as calmodulin, parvalbumin, calretinin, and calbindin, serve as essential buffers to protect neurons from calcium overload and excitotoxicity [161]. These proteins act as reservoirs for excess calcium, helping to stabilize intracellular concentrations and preserve neuronal function under conditions of calcium dysregulation. For instance, calmodulin plays a pivotal role in regulating ion channels, particularly by modulating sodium and potassium currents, which are crucial in controlling neuronal excitability. Overexpression of calmodulin has been shown to restore potassium currents and suppress hyperactivity in neurons, suggesting its therapeutic potential in epilepsy treatment [162]. Parvalbumin, which is highly expressed in GABAergic interneurons, is vital for inhibiting excitatory signals in the brain. A decrease in parvalbumin-positive interneurons has been observed in various forms of epilepsy, including temporal lobe epilepsy (TLE) and hippocampal sclerosis, where synaptic remodeling contributes to seizure activity [18]. Studies have demonstrated that modulating parvalbumin-positive interneurons through pharmacogenetic approaches can significantly delay seizure onset and reduce seizure severity, making them a promising target for therapeutic interventions [163]. Similarly, calretinin, another CaBP primarily involved in inhibitory networks, has been found to decrease in epileptic tissues, and its loss may exacerbate excitatory neuronal activity, contributing to seizure development [164]. Maintaining the integrity of calretinin-positive cells and their connections with principal cells may have protective effects against epilepsy. Calbindin, another member of the CaBP family, plays a key role in maintaining intracellular calcium homeostasis and has been linked to hippocampal function, particularly in the dentate gyrus. Its expression is significantly reduced in patients with epilepsy, including those with TLE and Ammon’s horn sclerosis (AHS), which contributes to impaired calcium handling and neuronal hyperexcitability [165,166]. Additionally, calbindin helps inhibit apoptosis and endoplasmic reticulum (ER) stress pathways, both of which are implicated in the pathogenesis of epilepsy [167]. Strategies that restore calbindin expression or function, such as cell grafting, have shown potential in reducing hyperexcitability and seizure activity in animal models [168]. In summary, Ca^2+^ and calcium-binding proteins are deeply intertwined with the molecular mechanisms underlying epilepsy. By buffering excess calcium and modulating ion channels, CaBPs like calmodulin, parvalbumin, calretinin, and calbindin play critical roles in both the prevention and management of epileptic seizures. These proteins not only protect neurons from excitotoxicity but also provide therapeutic targets for developing novel treatments aimed at stabilizing calcium homeostasis and reducing seizure susceptibility. Future research should focus on therapies targeting calcium dysregulation in epilepsy, particularly through the modulation of CaBPs. Enhancing calmodulin function could restore potassium currents and reduce neuronal hyperactivity, offering therapeutic potential for epilepsy treatment [169,198]. Additionally, targeting parvalbumin-positive interneurons through gene therapy or pharmacogenetic tools could reduce seizure severity [169]. Restoring calretinin and calbindin expression in hippocampal regions may also improve calcium homeostasis and reduce neuronal excitability [198]. Nanotechnology-based drug delivery systems could further improve the precision and efficacy of these treatments, stabilizing calcium signaling and reducing seizure susceptibility.

### 5.5. Calcium Ions as Diagnostic and Therapeutic Target in Migraine

Ca^2+^ play a significant role in both the pathophysiology and treatment of migraines, particularly through their involvement in calcium channels and related mechanisms (Table 1). Current migraine therapies include both prophylactic agents, such as antiepileptic drugs, and acute treatments like triptans, although many patients still rely on nonsteroidal anti-inflammatory drugs (NSAIDs) despite their limited efficacy. Unfortunately, a large percentage of migraine sufferers do not respond to these treatments, which underscores the need for more effective therapeutics [199,200,201]. Calcium channels, particularly CaV2.1, have been identified as potential therapeutic targets for migraine, especially in patients withFHM1. While there are no selective small molecule inhibitors for CaV2.1, peptide toxins like ω-agatoxin IVA and IVB have shown specificity for this channel, although they are not widely used clinically. Drugs such as flunarizine and verapamil, which also affect CaV2.1, have demonstrated clinical efficacy in some migraine patients [202]. Additionally, acid-sensing ion channels (ASICs), which enhance calcium permeability and are involved in pain signaling, represent another promising target for migraine therapy. ASIC1 channels, in particular, have been implicated in chronic inflammatory and neuropathic pain and are overexpressed in key brain regions associated with migraine. Blockers of ASIC1a, such as amiloride and the tarantula toxin PcTx1, have been shown to inhibit CSD, a key event in migraine pathophysiology, suggesting that targeting ASICs may offer a novel approach to migraine treatment [170,171,172]. Future research should focus on developing more effective treatments for migraines by targeting calcium channels and related mechanisms. Selective inhibitors of CaV2.1, such as small molecules or peptide toxins like ω-agatoxin, may offer promising alternatives to current therapies, especially for patients with FHM1 [173,174]. Additionally, targeting ASICs, particularly ASIC1a, could provide a novel therapeutic approach. Compounds like amiloride or PcTx1 that block ASIC1a have shown promise in inhibiting cortical CSD, a key event in migraine pathophysiology [174]. Exploring these targets further, alongside the development of more specific, accessible drugs, could lead to more effective migraine therapies.

## 6. Conclusions

Ca^2+^ are integral to the functioning of the CNS, influencing everything from neurotransmitter release to synaptic plasticity and overall neuronal excitability. Through their role as second messengers in signaling cascades, Ca^2+^ contribute to a wide array of physiological processes essential for normal brain function, including learning, memory, and synaptic modulation. The tight regulation of calcium homeostasis is critical, as even minor disruptions can lead to profound changes in neuronal function, highlighting the central importance of Ca^2+^ in maintaining neural health and communication.

To advance our understanding of these complex mechanisms, it is crucial for future research to continue exploring the significance of Ca^2+^ in CNS function and dysfunction. Investigating the intricate pathways involving calcium signaling could lead to novel therapeutic strategies and improved outcomes for patients affected by neurological conditions. Encouraging interdisciplinary studies and innovative methodologies will be key in uncovering the full impact of Ca^2+^ on brain health.
